# Winter Quarterly Report on the Diseases of Cincinnati, Concluded—Treatment of Bronchitis

**Published:** 1828-05

**Authors:** 


### III. ORIGINAL.

5. *Winter Quarterly Report on the Diseases of Cincinnati, concluded.* In our last number we spoke of Bronchitis, as constituting the predominant disease of the past winter. We must now say, that it was not the only form of thoracic inflammation which occurred among us. Cases of Croup, Pleurisy and Pneumonia were, occasionally, met with, both distinct from Bronchitis and associated with that malady; but they were, comparatively, rare. Indeed, according to our own observations, the pulmonary diseases of Cincinnati have, in these lat- years, undergone a change, in their relative frequency; so that Croup and Pleurisy are less frequent, while Bronchitis is more common, than in former years. We do not pretend to have discovered the cause of this variation; but, at present, feel assured of its reality. Perhaps the denser population of the city; the establishment of a great variety of manufactures; the erection of more than twenty stationary steam engines; and the establishment of half that number of foundries, in all of which the combustion of a stone-coal, abounding in bitumen and sulphur, takes place to a great extent; finally, the multiplication of stone-cutters yards, where large quantities of friable sand-stone are sawed, cut and polished, in which operations clouds of arenaceous dust are sent abroad, may have given to the city a factitious atmosphere, which has deranged, to a certain degree, the vital properties of the Bronchial membrane, and predisposed it to the action of vicissitudes of temperature. If this can explain the greater frequency of Bronchitis, it will, at the same time, account for the comparative rarity of other forms of pulmonary disease; for the influence of atmospheric changes being invited upon the mucous membrane of the lungs, their other tissues would, of course, in a great degree, escape.

*Treatment of Bronchitis and other Pectoral Inflammations.* It is not our design to enter fully into this subject. We shall limit ourselves to a few remarks on the more important remedies, and first of BLOODLETTING. It would be superfluous to urge upon the profession the necessity of early, copious, and, in many cases, repeated bloodletting, in acute inflammations of the serous membranes of the chest and lungs; or to insist on the value of cupping, in those of a chronic character; for every physician is aware of the importance of these remedies, in such cases. But in Bronchitis both acute and chronic, venesection is often omitted to the great, if not fatal, injury of the patient. It is not difficult to perceive the causes of this omission. In the first place, many cases of bronchial inflammation are unattended with pain or a tense pulse; two circumstances which tend, as powerfully as negatives can tend, to divert the mind from venesection. Secondly, when the disease is accompanied with pleuritic pain, it is, also, attended with expectoration, which is apt to suggest to the physician, that it is a case of pleurisy or pneumonia, in the expectorating stages, when bloodletting is, in general, contra-indicated. Thirdly, bronchitis very frequently attacks infants and aged persons who are *cæteris paribus*, much less frequently and copiously bled, than those in middle life. From the influence of these causes many patients have been lost, who might, by the judicious employment of the remedy under consideration, have been saved. As every thing, however, has its limits, so it is possible, in bronchial inflammation, to carry venesection too far. Of this abuse we have met with several instructive examples. When the disease has existed for several weeks, and is attended with copious expectoration, the powers of the system are apt to fail, from the further loss of blood, and the patient to sink under the copious secretion, almost passive, which is going on from an extensive surface. *Tartarized Antimony.* We have found this medicine of great value in Bronchitis. It may be adapted to every stage. In the early and acute periods of the disease, it may be administered in liberal quantities, alone, or combined with nitrate of potash, so as to nauseate, heavily, and excite full vomiting; which never fails to give signal relief, provided bloodletting has been premised. In more advanced stages it may be conjoined with Opium in pills, or laudanum may be added to a solution of the tartrate. *Calomel and Squills.* We have employed this combination with much advantage in Bronchitis. When the disease is not extremely acute, the cure, after a single bloodletting, may be commenced, with this compound; and in mild and chronic forms we have often, with success, made it the first remedy. It should be administered, for two or three days, in such quantities, as to nauseate the patient; and, by the end of that time, to have produced some effect on the mouth. It may, then, be followed by gentler measures. *Digitalis.* This is an important remedy, in the more chronic cases. When the inflammatory action is considerable, we give in connexion with tartarized antimony, in a dilute solution. The following formula, on the whole, is the best we have employed:

| | |
|---|---|
| ℞ Tartarized antimony, | gr. ij. |
| Saturated tincture of digitalis, | ʒij. |
| Spirit of nitros ether, | ʒvj. |
| Mucilage of gum arabic, with distilled water, ℥vij. Mix. | |

A table spoonful (℥ss) to be given every 2, 4, 6 or 8 hours, according to circumstances; and repeated 'till the pulse experiences the characteristic power of the digitalis. In advanced stages of the disease, the tincture of opium should be added to the solution, in such quantities as to keep the patient slightly under its action. We have met with cases which required the long continued administration of digitalis. In many of these, we have united the tincture, with the compound tincture of Benzion, so long celebrated among the people, by the name of Balsam of Life, and used and abused in pectoral maladies. The combination of which we speak, diminishes expectoration, allays morbid irritability, and strengthens the lungs, in a more decided manner than any other medicine with which we are acquainted. Of the *Colchicum* we have already expressed our opinion at p. 94 of this number. The *Bark*. There are stages of bronchial inflammation which call for this medicine. In phlegmatic temperaments, when the disease has existed for some time, the expectoration is profuse, and the pulse feeble and frequent, the Bark may be administered with great effect. Opium, under these circumstances, is an excellent adjuvant. The pulse speedily becomes slower and fuller; the expectoration diminishes; and the cough, which is kept up by the irritation of secreted mucus, consequently abates; while all the symptoms are aggravated, even to a fatal degree, under a debilitating plan. *External applications*. We have not found blisters or tartarized plasters, so beneficial in bronchitis, as many of our brethren suppose them; if we may judge from the frequency with which a resort is had to them. When pleuritic pain exists, they are useful; but in the absence of this, we have not often seen them effect much. It is necessary, however, to attend to the state of the skin, which is often unhealthy. In feeble constitutions, when the disease is of a subacute character, the cutaneous circulation is defective, especially in the lower extremities, and the feet are apt to be cold. The perspiration is of course suspended, and the lungs are in a state of congestion. This condition, occasionally, requires bloodletting; and when that remedy is beneficial, it is always followed by increased energy of the pulse. The greater number of cases, however, do not require, or even admit of, venesection. In all of them a hot salt, foot-bath, of the strength of a pint of common salt to the gallon of water, used twice or thrice in the course of twenty-four hours, with sinapisms to the soles of the feet, and, in some cases, blisters to the ankles, is attended with the happiest effects. After two or three days, the heat becomes equally distributed, the perspiratory function is restored in an equal degree, the determination upon the mucous membrane is diminished, and the dyspnœa of the patient sensibly mitigated. With these remarks we dismiss the subject.

